# Incidence of Access to Ambulatory Mental Health Care Prior to a Psychiatric Emergency Department Visit Among Adults in Ontario, 2010-2018

**DOI:** 10.1001/jamanetworkopen.2021.5902

**Published:** 2021-04-14

**Authors:** Paul Kurdyak, Sima Gandhi, Laura Holder, Mohammed Rashid, Natasha Saunders, Maria Chiu, Astrid Guttmann, Simone Vigod

**Affiliations:** 1 ICES, Toronto, Ontario, Canada; 2 Department of Psychiatry, Temerty Faculty of Medicine, University of Toronto, Toronto, Ontario, Canada; 3 Institute of Health Policy, Management and Evaluation, Dalla Lana School of Public Health, Temerty Faculty of Medicine, University of Toronto, Toronto, Ontario, Canada; 4 Centre for Addiction and Mental Health, Toronto, Ontario, Canada; 5 Hospital for Sick Children, Toronto, Ontario, Canada; 6 Department of Pediatrics, Temerty Faculty of Medicine, University of Toronto, Toronto, Ontario, Canada; 7 Women’s College Hospital, Toronto, Ontario, Canada

## Abstract

**Importance:**

Psychiatric emergency department (ED) visits may be avoidable if individuals have access to adequate outpatient care, but the extent to which individuals use the ED itself as a key point of access is largely unknown.

**Objective:**

To describe the extent to which the ED is a first point of contact for mental health care among adults, and identify key factors associated with this outcome.

**Design, Setting, and Participants:**

This cohort study was conducted from 2010 to 2018 in Ontario, Canada. Using health care administrative data, all individuals aged 16 years and older with an incident psychiatric ED visit were included. Statistical analysis was performed from September 2019 to February 2021.

**Main Outcomes and Measures:**

The primary outcome was no outpatient mental health or addictions contact in the 2 years preceding the incident ED visit. The study also measured predisposing (age, sex, immigrant status, comorbidity), enabling (neighborhood income, rurality, continuity of primary care), and need factors (diagnosis from incident ED visit) associated with the so-called first-contact ED visits, generating adjusted odds ratios (aORs) and 95% CIs.

**Results:**

The cohort included 659 084 patients who visited the ED. Among these patients, 340 354 were female individuals (51.6%), and the mean (SD) age was 39.1 (18.5) years. The incident ED visit was a first contact for 298 924 individuals (45.4%). Patients who had increased odds of first-contact ED visits included older individuals (aged 65-84 years vs 16-24 years; aOR, 1.13; 95% CI, 1.12-1.14), male individuals (aOR, 1.14; 95% CI, 1.13-1.15), immigrants (eg, economic class immigrant vs nonimmigrant status: aOR, 1.20; 95% CI, 1.18-1.21), rural residents (aOR, 1.21; 95% CI, 1.20-1.21), and in those with minimal primary care (aOR, 1.68; 95% CI, 1.67-1.69). All diagnoses had a higher likelihood of first-contact ED visits than mood disorders, particularly substance and alcohol use disorders (aOR, 1.66; 95% CI, 1.65-1.68).

**Conclusions and Relevance:**

This study’s results suggest that nearly half of incident psychiatric ED visits were first-contact visits, which may be avoidable. Targeted efforts to improve outpatient access should focus on populations with risk factors for first-contact ED presentations, including men, older adults, rural residents, those with poor primary care connectivity, and those with substance-related diagnoses.

## Key Points

**Question:**

What proportion of individuals aged 16 years and older seeking care at an emergency department (ED) for mental health or addiction-related reasons have had no access to outpatient services in the preceding 2 years?

**Findings:**

In this cohort study, 659 084 adults had a first ED visit, among whom 298 924 (45.4%) had no prior outpatient contact. Characteristics associated with poor access to mental health care generally, such as male sex, living in a rural area, presenting with a substance use disorder, and no usual primary care clinician, were associated with a first ED visit for mental health or addiction-related reasons with no prior outpatient contact.

**Meaning:**

Nearly half of incident psychiatric ED visits were first-contact visits, which may have been avoidable with better access to outpatient mental health and addiction services.

## Teaser

This cohort study estimates the proportion of individuals aged 16 years and older in Ontario, Canada, with incident psychiatric emergency department (ED) visits who have not previously accessed outpatient mental health or addiction services in the prior 2 years.

## Introduction

Emergency department (ED) use for mental illness and addiction is increasing.^1,2,3^ The increasing ED visit volumes for mental illness and addiction suggests that the ED is an important access point. The ED may be necessary for high acuity presentations, particularly those with very rapid onset or where safety is a primary concern. However, it is likely not the ideal service site for most individuals seeking help for mental health and addiction (MHA) issues. Patients would be better served in outpatient settings, with access occurring in a timely fashion. Access to mental health services, and particularly psychiatrists, is often limited, and when available, is associated with long wait times.^4,5^

In previous studies, more than half of children and youth had no prior contact with outpatient mental health services in the 2 years prior to their first psychiatric ED visit,^6^ and for women postpartum, the rate of first contact was as high as 60%.^7^ Whether high rates of first contact in the ED are the norm in general adult populations is largely unknown.

The main objectives of this study were to describe the proportion of individuals aged 16 years and older who had no outpatient MHA-related contact in the 2 years preceding an incident ED visit (hereafter referred to as a first-contact ED visit) for a mental illness or addiction, and to identify the sociodemographic and clinical characteristics associated with the ED being a first point of contact. We hypothesized that the proportion of individuals with a first contact ED visit would be just as high (>50%) as what was previously observed in children and youth.^6^

## Methods

### Study Design and Setting

Data for this cohort study were obtained from population-based administrative health data sets housed at ICES, a not-for-profit research institute encompassing a community of research, data and clinical experts, and a secure and accessible array of Ontario's health-related data, allowing linkage of individual health records across databases using encoded identifiers. The use of data in this project was authorized under section 45 of Ontario’s Personal Health Information Protection Act, which does not require review by a research ethics board nor informed patient consent. This study followed the Strengthening the Reporting of Observational Studies in Epidemiology (STROBE) reporting guideline.

### Data Sources

This study uses health administrative data from Ontario, Canada’s most populous province with 14 million people representing approximately 40% of the Canadian population. In Ontario, most hospital and physician services are funded through the single payer Ontario Health Insurance Plan (OHIP).

The study cohort was developed using diagnostic codes from ED records (National Ambulatory Care Reporting System), using the *International Classification of Diseases and Related Health Problems, Tenth Revision, Canada (ICD-10-CA)*. Mental health and addiction-related outpatient visits were obtained using information from the OHIP physician billings database. Hospitalization information was obtained using inpatient discharge records in the Discharge Abstract Database (DAD), and from the Ontario Mental Health Reporting System (OMHRS).

Comorbidity was calculated using the Adjusted Clinical Group (ACG) scoring system, a case-mix adjustment system based on individual characteristics and diagnoses received in outpatient and inpatient settings in the previous year. Diagnostic codes were grouped into 32 Aggregated Diagnostic Groups (ADGs), similar in terms of clinical characteristics, health service use, and resource needs.^8^ We excluded 3 psychosocial ADGs representing psychiatric comorbidity because our ED cohorts were all based on mental health diagnoses. Previous studies have shown the ADGs are an excellent estimator of mortality in a general population^9^ and in a population of individuals with a diagnosis of schizophrenia.^10^

The Registered Persons Database (RPDB) includes demographic information for all Ontarians eligible for public health insurance, and was used to obtain age, sex, and postal code. Geography, defined as urban or rural (communities with 10 000 residents or less), and neighborhood income quintile for each census dissemination area were obtained using postal codes linked to the 2011 Canadian Census. Immigrant status (family class immigrant, economic immigrants, resettled refugees, other) was obtained using the Immigration, Refugees and Citizenship Canada’s (IRCC) Permanent Resident database, which includes immigration records for individuals who have permission to live and stay in Ontario since 1985.^11^

### Study Population

The cohort included Ontario adults and youths, aged 16 and older, presenting to the ED for the first time for mental health and addictions (MHA)-related issues, between April 1, 2010, and March 31, 2018. A first MHA-related ED visit was defined as having no MHA-related ED visits or hospital admissions in the 2 years prior to the incident MHA-related ED visit. We excluded individuals older than 105 years of age, non-Ontario residents, those with a missing or invalid health card number, and those without OHIP eligibility for at least 2 years prior to the incident ED visit to ensure we were able to measure prior outpatient mental health care. Planned or scheduled ED visits were also excluded because these visits are often primary care visits that happen to occur in ED settings, most frequently in rural regions where primary care physicians work in both ED and outpatient settings.

### Measures

#### Main Outcome

The primary outcome was a first-contact ED visit in the mental health care system, defined as an incident MHA-related ED visit, without previous MHA care in an outpatient setting in the preceding 2 years. MHA care in outpatient settings was defined as any visit to a psychiatrist or a visit to a primary care physician with specific outpatient visit claims validated as mental health-related.^12^ A larger proportion of individuals for whom the MHA-related ED visit is a first contact with the health care system reflects poorer access to outpatient care, a reduced propensity to seek care, or both.

#### Covariates

We used the Andersen-Newman comprehensive model of societal and individual determinants of health care use,^13^ which proposes that health care access and use are a function of 3 characteristics: predisposing factors; enabling factors, and need factors. Predisposing factors include sociocultural factors that were present prior to illness, for example, age, sex, health beliefs, and social structure. Enabling factors include the logistical aspects of obtaining or accessing care, such as income, availability of resources, and quality of social relationships. Need factors are the most immediate reasons for seeking health services, including health and functional issues. The Andersen-Newman model is a useful model to serve as a framework for evaluating the factors associated with our outcome, first ED contact, which is a health care access measure.

Predisposing factors in this study included age, sex, immigrant status, and the number of comorbid conditions (because individuals with greater comorbidity are more likely to visit EDs). Enabling factors included neighborhood income quintile, rurality (urban, rural), and whether or not individuals had a usual clinician of primary care at the time of the incident ED visit. Usual clinician of primary care was defined based on whether patients were rostered to a family physician in a primary care model.

Need factors included information related to the reason for health service use, obtained from the most responsible diagnosis on the incident ED record. Mental health conditions included substance-related disorders, schizophrenia, and other nonorganic psychotic disorders (hereafter referred to as psychotic disorders), mood disorders, anxiety and adjustment disorders, self-harm, and other, as has been used in previous publications^6^ (*ICD-10* codes listed in eTable 1 in the Supplement). Self-harm was a mutually exclusive category; patients had a self-harm MHA-related visit if the ED visit was for self-harm and there was no other MHA-related diagnosis. The severity of the incident ED visit was examined using the Canadian Triage Acuity Scale (CTAS),^14^ which describes the severity of patients. Scores between 1 and 3 represent high-acuity or urgent, whereas scores of 4 and 5 are considered nonurgent. Finally, we studied whether the incident ED visit resulted in a hospital admission or discharge home.

### Statistical Analysis

We described the study cohort at baseline, defined as the date of the incident MHA-related ED visit, comparing individuals based on the presence or absence of previous outpatient mental health visit in the previous 2 years. We also conducted exploratory analyses by replicating the baseline descriptive and outcome analyses within each of the 4 diagnostic groups previously listed, as well as within those with a self-harm diagnosis (in any field) and those without any self-harm diagnosis. To evaluate the association between the predisposing, enabling, and need factors of the Andersen-Newman model, we developed exploratory logistic regression models. The absence of prior outpatient contact (ED visit as first contact) was our dependent variable. Our modeling approach was to first include predisposing factors, then to include enabling factors, and then to include need factors. These sequential models were conducted in the entire cohort and for the diagnostic subsets. Confidence intervals were included to allow readers to determine whether the regression estimates were statistically significant. The 95% CIs were calculated based on the asymptotic χ^2^ distribution of the generalized likelihood ratio test. Analyses were performed using SAS Enterprise Guide version 7.1 (SAS Institute) from September 2019 to February 2021.

## Results

Among 659 084 individuals with an incident ED visit, 340 354 were female individuals (51.6%), and the mean (SD) age was 39.1 (18.5) years. Of all incident ED visits, 298 924 were first-contact visits (45.4%), and 15.6% (102 586) resulted in hospitalization. Among 298 924 first-contact visits, 11.7% (34 902) resulted in hospitalization, whereas among 360 160 visits with prior MHA contact, 18.8% (67 684) resulted in hospitalization (Table 1). There were fewer than 506 individuals (0.1%) who died during the ED visit (eFigure in the Supplement).

**Table 1.** Characteristics of Adults With a First Contact Emergency Department Visit by Prior Outpatient Care for Mental Health and Addictions-Related Disorders, 2010-2018

Variable	Patients, No. (%)		
	Total (N=659 084)	Prior MHA contact (n=360 160)	First contact (n=298 924)
Predisposing factors			
Age, mean (SD), y	39.13 (18.46)	39.86 (17.89)	38.24 (19.08)
Age group, y			
16-24	192 988 (29.3)	93 211 (25.9)	99 777 (33.4)
25-34	125 441 (19.0)	68 981 (19.2)	56 460 (18.9)
35-44	99 837 (15.1)	59 744 (16.6)	40 093 (13.4)
45-54	101 293 (15.4)	61 469 (17.1)	39 824 (13.3)
55-64	68 210 (10.3)	40 014 (11.1)	28 196 (9.4)
65-84	60 963 (9.2)	31 984 (8.9)	28 979 (9.7)
≥85	10 352 (1.6)	4757 (1.3)	5595 (1.9)
Sex			
Female	340 359 (51.6)	201 929 (56.1)	138 430 (46.3)
Male	318 725 (48.4)	158 231 (43.9)	160 494 (53.7)
Immigration category			
Nonimmigrants	582 623 (88.4)	321 125 (89.2)	261 498 (87.5)
Resettled refugees	17 894 (2.7)	9099 (2.5)	8795 (2.9)
Family class immigrants	26 988 (4.1)	14 070 (3.9)	12 918 (4.3)
Economic class immigrants	30 124 (4.6)	15 162 (4.2)	14 962 (5.0)
Other immigrants	1455 (0.2)	704 (0.2)	751 (0.3)
No. of comorbid conditions, mean (SD)	5.81 (3.60)	6.42 (3.61)	5.08 (3.44)
Enabling factors			
Income quintile			
Missing	3755 (0.6)	1635 (0.5)	2120 (0.7)
Q1 (lowest)	175 278 (26.6)	95 331 (26.5)	79 947 (26.7)
Q2	139 354 (21.1)	76 271 (21.2)	63 083 (21.1)
Q3	123 876 (18.8)	67 418 (18.7)	56 458 (18.9)
Q4	113 522 (17.2)	62 165 (17.3)	51 357 (17.2)
Q5 (highest)	103 299 (15.7)	57 340 (15.9)	45 959 (15.4)
Rurality			
Missing	1336 (0.2)	673 (0.2)	663 (0.2)
Urban	563 464 (85.5)	317 355 (88.1)	246 109 (82.3)
Rural	94 284 (14.3)	42 132 (11.7)	52 152 (17.4)
Usual clinician of care (% yes)	620 262 (94.1)	355 495 (98.7)	264 767 (88.6)
Need factors			
MHA diagnosis at index ED visit			
Substance-related disorders	152 641 (23.2)	67 389 (18.7)	85 252 (28.5)
Schizophrenia and other nonorganic psychotic disorders	35 819 (5.4)	23 274 (6.5)	12 545 (4.2)
Mood disorders	117 638 (17.8)	82 281 (22.8)	35 357 (11.8)
Anxiety and adjustment disorders	251 236 (38.1)	132 757 (36.9)	118 479 (39.6)
Other mental health disorders	101 750 (15.4)	54 459 (15.1)	47 291 (15.8)
Self-harm diagnosis at index ED visit	56 164 (8.5)	31 271 (8.7)	24 893 (8.3)
Acuity (CTAS) score at index ED visit			
High acuity	543 468 (82.5)	302 847 (84.1)	240 621 (80.5)
Low acuity	113 838 (17.3)	56 501 (15.7)	57 337 (19.2)
Admission to inpatient care from index ED visit	102 586 (15.6)	67 684 (18.8)	34 902 (11.7)
Death during index ED visit	506 (0.1)	245 (0.1)	261 (0.1)

Abbreviations: CTAS, Canadian Triage Acuity Scale; ED, emergency department; MHA, mental health and addiction.

Patients with first-contact ED visits were younger than those with prior MHA contact (median [interquartile range {IQR}] age, 33 [22-51] years vs 37 [24-52] years), more likely to be male (160 494 [53.7%] vs 158 231 [43.9%]), more likely to live in a rural setting (52 152 [17.4%] vs 42 132 [11.7%]), and more likely to have no usual clinician of primary care (34 157 [11.4%] vs 4655 [1.3%]). Neighborhood income was similar between groups. First contact ED visit patients were more likely to have a low-acuity incident ED visit (57 337 individuals [19.2%] vs 56 501 individuals [15.7%]). Self-harm at the time of the incident ED visit were similarly prevalent between groups (first contact: 24 893 individuals [8.3%] vs prior MHA contact: 31 271 individuals [8.7%]).

First-contact ED visits, as a proportion of all incident ED visits, varied by diagnosis (Figure), with the lowest rates of first contact occurring among individuals presenting with mood disorders and the highest rate occurring among individuals with substance-related ED visits. There was no difference in rate of first contact between individuals with and without self-harm as part of their MHA ED visit (24 893 individuals with self-harm [44.3%] vs 274 031 individuals without self-harm [45.4%]) (Figure). Descriptive characteristics by first contact status for individuals with psychotic disorders or mood disorder-related ED visits (eTable 2 in the Supplement), anxiety and adjustment disorder or substance-related ED visits (eTable 3 in the Supplement), and individuals with and without self-harm-related ED visits (eTable 4 in the Supplement) are available in in the Supplement.

**Figure.**
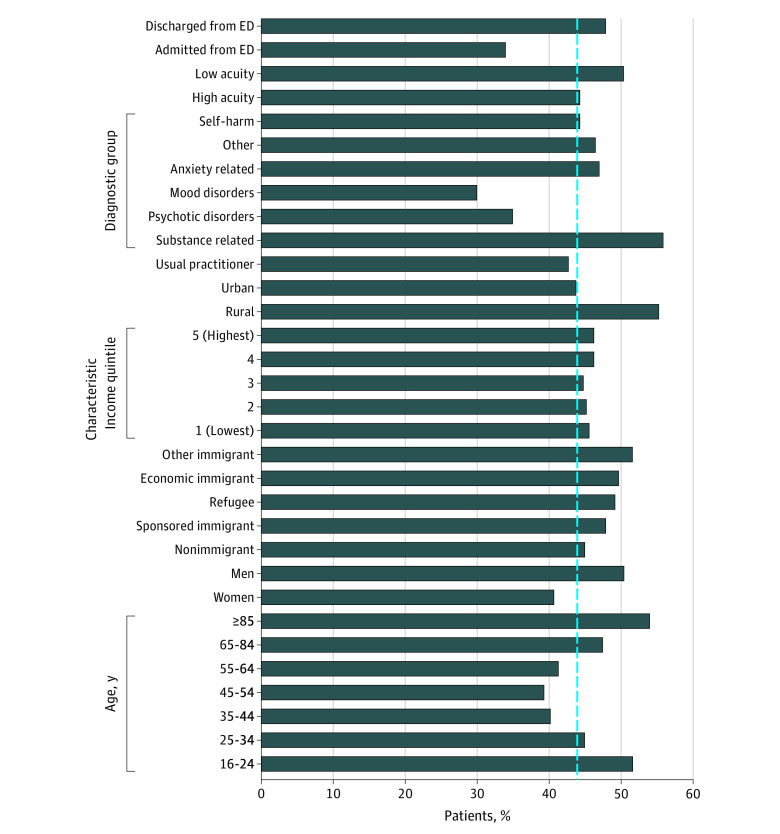
Emergency Department (ED) Visits With No Prior Outpatient Care for Mental Health and Addictions-Related Disorders by Predisposing, Enabling, and Need Factors, 2010-2018 Dotted line denotes overall ED visits with no prior outpatient care for mental health and addiction-related disorders (45.4% [298 924 of 659 084 visits]).

From the fully adjusted regression model (model 3 in Table 2), the predisposing factors associated with a greater likelihood of having a first-contact ED visit were older age (65-84 years vs 16-24 years: adjusted odds ratio [aOR], 1.14; 95% CI, 1.13-1.15; aged ≥85 years vs 16-24 years: aOR, 1.40; 95% CI, 1.37-1.42), male sex (vs female sex: aOR, 1.14; 95% CI, 1.13-1.14), and immigrant status (economic class immigrant vs nonimmigrant status: aOR, 1.20; 95% CI, 1.18-1.21; sponsored family class immigrant vs nonimmigrant status: aOR, 1.21; 95% CI, 1.20-1.23; other immigrant vs nonimmigrant status: aOR, 1.25; 95% CI, 1.19-1.31; resettled refugee vs nonimmigrant status: aOR, 1.20; 95% CI, 1.18-1.21). In terms of enabling factors, neighborhood income level was not a significant factor, but living in a rural region was associated with a higher likelihood of a first contact visit (aOR, 1.20; 95% CI, 1.20-1.21), as was having no usual care clinician (aOR, 1.68; 95% CI, 1.67-1.69). Among need factors, high acuity level at incident ED visit (aOR, 0.94; 95% CI, 0.94-0.95) and hospitalization at incident ED visit (aOR, 0.83; 95% CI, 0.82-0.84) were both associated with lower likelihood of a first-contact visit. Compared with mood disorders, all other diagnostic categories were associated with a higher likelihood of a first contact visit (psychotic disorders: aOR, 1.13; 95% CI, 1.12-1.15; anxiety and adjustment disorders: aOR, 1.51; 95% CI, 1.49-1.52; substance-related disorders: aOR, 1.66; 95% CI, 1.65-1.68; and other disorders: aOR, 1.53; 95% CI, 1.51-1.55). Self-harm at incident ED visit was not significantly associated with prior MHA care.

**Table 2.** aORs of No Prior Outpatient Care for Mental Health and Addictions-Related Emergency Department Visits, 2010-2018

Variable	aOR (95% CI)
Model 1: Predisposing factors	
Age, y	
16-24	1 [Reference]
25-34	0.88 (0.88-0.89)
35-44	0.81 (0.80-0.82)
45-54	0.81 (0.81-0.82)
55-64	0.89 (0.88-0.90)
65-84	1.14 (1.13-1.15)
≥85	1.40 (1.38-1.43)
Male (reference = female)	1.14 (1.13-1.14)
Immigration category	
Nonimmigrant	1 [Reference]
Economic class immigrants	1.14 (1.13-1.15)
Other immigrants	1.22 (1.16-1.28)
Resettled refugees	1.15 (1.14-1.17)
Family class immigrants	1.15 (1.14-1.17)
No. of comorbid conditions (per each additional condition)	0.94 (0.94-0.94)
Model 2: Predisposing + enabling factors	
Age, y	
16-24	1 [Reference]
25-34	0.86 (0.86-0.87)
35-44	0.79 (0.79-0.80)
45-54	0.79 (0.79-0.80)
55-64	0.87 (0.86-0.87)
65-84	1.10 (1.09-1.11)
≥85	1.35 (1.32-1.37)
Male (reference = female)	1.12 (1.12-1.13)
Immigration category	
Nonimmigrant	1 [Reference]
Economic class immigrants	1.20 (1.19-1.21)
Other immigrants	1.26 (1.20-1.32)
Resettled refugees	1.20 (1.19-1.22)
Family class immigrants	1.21 (1.19-1.22)
No. of comorbid conditions (per each additional condition)	0.95 (0.95-0.95)
Income quintile	
Q5 (highest)	1 [Reference]
Q1 (lowest)	1.01 (1.00-1.02)
Q2	1.02 (1.01-1.03)
Q3	1.03 (1.02-1.04)
Q4	1.02 (1.01-1.03)
Rural (reference = urban)	1.24 (1.23-1.25)
No UPC (reference = has usual provider of care)	1.71 (1.70-1.72)
Model 3: Predisposing + enabling + need factors	
Age, y	
16-24	1 [Reference]
25-34	0.87 (0.86-0.88)
35-44	0.81 (0.80-081)
45-54	0.81 (0.81-0.82)
55-64	0.89 (0.88-0.90)
65-84	1.13 (1.12-1.14)
≥85	1.40 (1.37-1.42)
Male (reference = female)	1.10 (1.09-1.10)
Immigration category	
Nonimmigrant	1 [Reference]
Economic class immigrants	1.20 (1.18-1.21)
Other immigrants	1.25 (1.19-1.31)
Resettled refugees	1.20 (1.18-1.21)
Family class immigrants	1.21 (1.20-1.23)
No. of comorbid conditions (per each additional condition)	0.95 (0.95-0.95)
Income quintile	
Q5 (highest)	1 [Reference]
Q1 (lowest)	1.01 (1.00-1.02)
Q2	1.02 (1.01-1.03)
Q3	1.03 (1.02-1.04)
Q4	1.02 (1.01-1.03)
Rural (reference = urban)	1.21 (1.20-1.21)
No UPC (reference = has usual clinician of care)	1.68 (1.67-1.69)
High acuity index ED visit (reference = low acuity)	0.94 (0.94-0.95)
Admitted from ED (reference = not admitted)	0.83 (0.82-0.84)
MHA diagnosis at index ED visit	
Mood and affective disorders	1 [Reference]
Substance related disorders	1.66 (1.65-1.68)
Schizophrenia and other non-organic psychotic disorders	1.13 (1.12-1.15)
Anxiety and adjustment disorders	1.51 (1.49-1.52)
Other (unclassified)	1.53 (1.51-1.55)
Self-harm diagnosis at index ED visit	1.00 (0.98-1.01)

Abbreviations: aOR, adjusted odds ratio; ED, emergency department; MHA, mental health and addiction; UPC, usual clinician of primary care.

The models estimated within the diagnostic sub-categories and self-harm at incident ED visit (eTable 5 in the Supplement) had similar predisposing, enabling, and need factor estimates as the full cohort model with one exception. Among individuals with psychotic disorders, high acuity and hospitalization at first ED visit, both need factors, were associated with a higher likelihood of a first contact ED visit (eTable 5 in the Supplement).

## Discussion

Between 2010 and 2018, almost half (45.4%) of adults with a first MHA-related ED visit had no mental health outpatient care in the 2 years prior to the ED visit. This first-contact phenomenon was more likely in older individuals, male individuals, and immigrants. First contact was more common in those living in rural regions and in those with limited primary care connection. Only a small subgroup of individuals with first contact required psychiatric hospitalization (34 902 patients; 11.7%), and in general (ie, with the exception of those with psychotic disorders) these individuals presented with lower acuity of symptoms than those who presented with evidence of recent outpatient mental health contact. Although there are circumstances where use of the ED is justified, and therefore not a failure of access, these results suggest that the ED is being used for primary access to mental health services, and it is possible that these ED visits could have been avoided if individuals had alternatives to the ED for mental health care. It is concerning that it appears to be underserved populations—young and older individuals, men, rural residents with limited primary care connection—who are accessing the ED without prior mental health care. It is possible that these groups have a reduced propensity to seek care. Efforts to improve timely access to outpatient mental health care should target these groups.

The results from this study are similar to a study using the same first-contact construct in a child and youth population in Ontario,^6^ in which slightly more than half of all incident mental health-related ED visits were associated with no prior mental health-related outpatient contact. The factors associated with no prior mental health-related outpatient contact are consistent with survey data on mental health service use. For example, in the replication of the National Comorbidity Survey, being male, living in a rural area, and being of an underrepresented ethnicity (an approximation of immigration status) all estimated lower use of mental health services, whereas family income was not significantly associated with mental health service use.^15^ Similarly, age, sex, income, and presence of a mental disorder all associated with access to care in Canada, although there were important differences in determinants and rates of access across provinces.^16^ That the factors associated with a first ED contact with no prior outpatient mental health visits are similar to factors associated with access to care more generally is not surprising, but point to the first ED contact construct established in this study as a potentially useful indicator of mental health service access more generally.

The factors associated with high rates of the use of the ED for first contact for mental illness and addictions-related reasons are likely variable based on the population. Transitional age youth have access issues because they age out of child and youth services and need to transition into adult services. This transition can cause barriers to care,^17^ with recent evidence that transitional age youth who have continuous contact with primary care are more likely to successfully transition to adult services.^18^ Residents living in rural regions have far fewer outpatient mental health visits than urban residents,^19^ which may explain the high rate of the use of EDs for mental health care in rural regions. Individuals with substance-related disorders have very low rates of treatment.^20^ These populations all have challenges with outpatient care that would translate into a high rate of the use of an ED for first contact. Interventions to improve access in these populations should be population-specific. For example, telemedicine is a mode of service delivery that has the potential to improve access to care for rural communities.^21^ Finally, there are specific populations where poor access to care has known adverse consequences. The high acuity and high rate of hospitalization associated with first contact among individuals with a psychotic disorder diagnosis suggests treatment is delayed in this population. This is concerning given delays in treatment in this population are detrimental to clinical outcomes, including remission^22,23,24^ and mortality.^25,26^

### Limitations

This study has several limitations. Major limitations include the fact that data on nonphysician contact, including publicly funded mental health professionals (eg, social workers and psychologists) and privately funded mental health professionals were not available. The publicly funded mental health professionals are typically employed in primary care settings, EDs, or hospitals; it is unlikely that the lack of these data substantially biases our results. It is unknown whether access to privately funded mental health professionals prior to a first ED contact has resulted in a substantial overestimate of the rate of first ED contact. Additionally, the diagnostic categories used to stratify the sample have not been validated, but have been used previously to broadly categorize the services provided.^6,27^

## Conclusions

The ED is increasingly being used as a first point of contact for individuals with mental illnesses and addictions. Our study found that almost half of patients seeking care in the ED for mental illnesses and addictions have not accessed outpatient services in the 2 years prior. The ED is a reasonable resource of last resort, particularly in cases with high acuity or concerns for safety. Our findings suggest that the ED is being used as a replacement for outpatient services that could be accessed in a more timely fashion. In other words, a substantial proportion of the first-contact ED visits in our study appear to be avoidable. The factors associated with first-contact ED visits indicate that efforts to avoid the ED as a site of first contact require outpatient services to become more accessible.

## Notes

Supplement. **eFigure.** Flowchart Detailing Cohort Inclusion Criteria **eTable 1.** ICD-10 Codes Used for the Diagnostic Categories **eTable 2.** Characteristics of Adults With A First Contact Emergency Department Visit for Schizophrenia-Related or Mood Disorders, By Prior Outpatient Care for Mental Health and Addictions-Related Disorders, 2010-2018 **eTable 3.** Characteristics of Adults With A First Contact Emergency Department Visit for Anxiety or Substance and Substance-Related Disorders, By Prior Outpatient Care for Mental Health and Addictions-Related Disorders, 2010-2018 **eTable 4.** Characteristics of Adults With A First Contact Emergency Department Visit, By Prior Outpatient Care for Mental Health and Addictions-Related Disorders and Presence or Absence of Self-Harm Diagnosis, 2010-2018 **eTable 5.** Adjusted Odds Ratios of No Prior Outpatient Care for Mental Health and Addictions-Related Emergency Department Visits, By Diagnostic Category, 2010-2018
