# Supplementary material for: Incidence of Access to Ambulatory Mental Health Care Prior to a Psychiatric Emergency Department Visit Among Adults in Ontario, 2010-2018
Source: JAMA Netw Open. 2021 Apr 14;4(4):e215902. doi: 10.1001/jamanetworkopen.2021.5902 (PMC8047734; doi:10.1001/jamanetworkopen.2021.5902)
Supplement: Supplement. — eFigure. Flowchart Detailing Cohort Inclusion Criteria eTable 1. ICD-10 Codes Used for the Diagnostic Categories eTable 2. Characteristics of Adults With A First Contact Emergency Department Visit for Schizophrenia-Related or Mood Disorders, By Prior Outpatient Care for Mental Health and Addictions-Related Disorders, 2010-2018 eTable 3. Characteristics of Adults With A First Contact Emergency Department Visit for Anxiety or Substance and Substance-Related Disorders, By Prior Outpatient Care for Mental Health and Addictions-Related Disorders, 2010-2018 eTable 4. Characteristics of Adults With A First Contact Emergency Department Visit, By Prior Outpatient Care for Mental Health and Addictions-Related Disorders and Presence or Absence of Self-Harm Diagnosis, 2010-2018 eTable 5. Adjusted Odds Ratios of No Prior Outpatient Care for Mental Health and Addictions-Related Emergency Department Visits, By Diagnostic Category, 2010-2018 [file jamanetwopen-e215902-s001.pdf]

Supplemental Online Content

Kurdyak P, Gandhi S, Holder L, et al. Incidence of access to ambulatory mental health care prior to a psychiatric emergency department visit among adults in Ontario, 2010-2018. *JAMA Netw Open.* 2021;4(4):e215902. doi:10.1001/jamanetworkopen.2021.5902

eFigure. Flowchart Detailing Cohort Inclusion Criteria

eTable 1. ICD-10 Codes Used for the Diagnostic Categories

eTable 2. Characteristics of Adults With A First Contact Emergency Department Visit for Schizophrenia-Related or Mood Disorders, By Prior Outpatient Care for Mental Health and Addictions-Related Disorders, 2010-2018

eTable 3. Characteristics of Adults With A First Contact Emergency Department Visit for Anxiety or Substance and Substance-Related Disorders, By Prior Outpatient Care for Mental Health and Addictions-Related Disorders, 2010-2018

eTable 4. Characteristics of Adults With A First Contact Emergency Department Visit, By Prior Outpatient Care for Mental Health and Addictions-Related Disorders and Presence or Absence of Self-Harm Diagnosis, 2010-2018

eTable 5. Adjusted Odds Ratios of No Prior Outpatient Care for Mental Health and Addictions-Related Emergency Department Visits, By Diagnostic Category, 2010-2018

This supplemental material has been provided by the authors to give readers additional information about their work.

© 2021 Kurdyak P. *JAMA Network Open.*

eFigure 1. Flowchart detailing cohort inclusion criteria

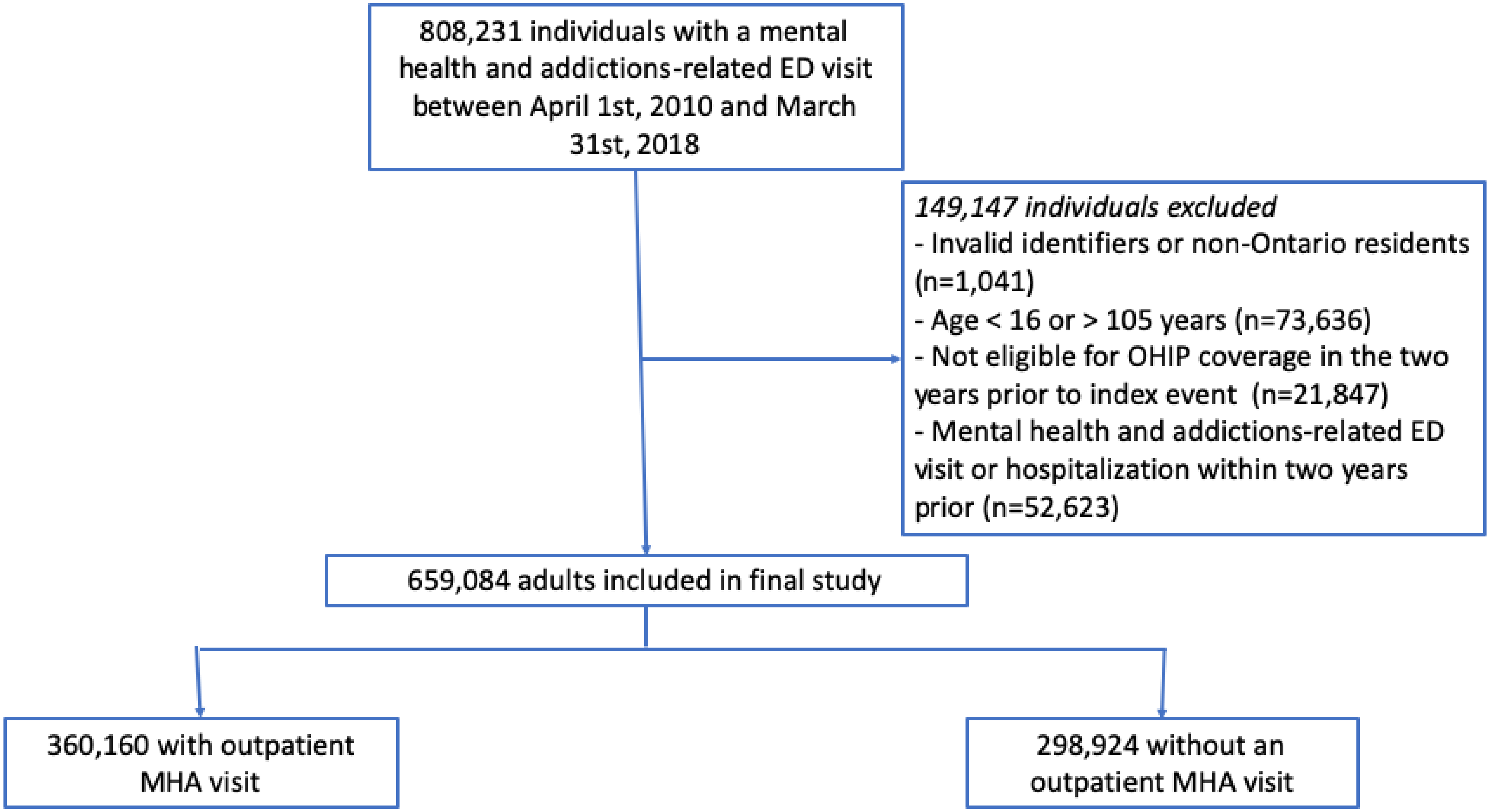

© 2021 Kurdyak P. *JAMA Network Open.*

eTable 1: ICD-10 codes used for the diagnostic categories.

**Diagnostic Category**	**ICD-10 Codes**
Substance-related Disorders	F55, F10-F19
Schizophrenia and other non-organic psychotic disorders	F20 (excluding F20.4), F22, F23, F24, F25, F28, F29, F53.1
Mood disorders	F30, F31, F32, F33, F34, F38, F39, F53.0
Anxiety and adjustment disorders	F40, F41, F42, F43, F48.8, F48.9; F93.1, F93.2

© 2021 Kurdyak P. *JAMA Network Open.*

eTable 2. Characteristics of adults with a first contact emergency department visit for schizophrenia-related or mood disorders, by prior outpatient care for mental health and addictions-related disorders, 2010-2018 (N=153,457).

	Schizophrenia and Other Non-Organic Psychotic Disorders			Mood Disorders		
**Variable**	**Total**	**Prior Contact**	**First Contact**	**Total**	**Prior Contact**	**First Contact**
**Adults with a first contact emergency department visit, N**	**35,819**	**23,274**	**12,545**	**117,638**	**82,281**	**35,357**
**Predisposing factors**						
**Age, mean ± SD**	42.96 ± 18.07	43.63 ± 17.41	41.73 ± 19.17	38.41 ± 18.15	39.38 ± 17.91	36.14 ± 18.48
**Age group (years), n(%)**						
16-24	6,896 (19.3%)	3,826 (16.4%)	3,070 (24.5%)	36,066 (30.7%)	22,860 (27.8%)	13,206 (37.4%)
25-34	6,777 (18.9%)	4,392 (18.9%)	2,385 (19.0%)	21,497 (18.3%)	14,663 (17.8%)	6,834 (19.3%)
35-44	6,008 (16.8%)	4,122 (17.7%)	1,886 (15.0%)	18,173 (15.4%)	13,382 (16.3%)	4,791 (13.6%)
45-54	6,727 (18.8%)	4,674 (20.1%)	2,053 (16.4%)	18,563 (15.8%)	14,181 (17.2%)	4,382 (12.4%)
55-64	4,699 (13.1%)	3,310 (14.2%)	1,389 (11.1%)	12,085 (10.3%)	9,197 (11.2%)	2,888 (8.2%)
65-84	4,025 (11.2%)	2,587 (11.1%)	1,438 (11.5%)	9,738 (8.3%)	7,068 (8.6%)	2,670 (7.6%)
85+	687 (1.9%)	363 (1.6%)	324 (2.6%)	1,516 (1.3%)	930 (1.1%)	586 (1.7%)
**Sex, n(%)**						
Female	14,790 (41.3%)	9,990 (42.9%)	4,800 (38.3%)	66,593 (56.6%)	49,270 (59.9%)	17,323 (49.0%)
Male	21,029 (58.7%)	13,284 (57.1%)	7,745 (61.7%)	51,045 (43.4%)	33,011 (40.1%)	18,034 (51.0%)
**Immigration category, n(%)**						
Non-immigrant	29,529 (82.4%)	19,359 (83.2%)	10,170 (81.1%)	105,929 (90.0%)	73,976 (89.9%)	31,953 (90.4%)
Family class immigrants	2,381 (6.6%)	1,496 (6.4%)	885 (7.1%)	4,264 (3.6%)	2,995 (3.6%)	1,269 (3.6%)
Resettled refugees	1,557 (4.3%)	972 (4.2%)	585 (4.7%)	2,451 (2.1%)	1,746 (2.1%)	705 (2.0%)
Other immigrants	129 (0.4%)	73 (0.3%)	56 (0.4%)	208 (0.2%)	144 (0.2%)	64 (0.2%)
Economic class immigrants	2,223 (6.2%)	1,374 (5.9%)	849 (6.8%)	4,786 (4.1%)	3,420 (4.2%)	1,366 (3.9%)
**Number of comorbid conditions, mean ± SD**	4.76 ± 3.61	5.26 ± 3.65	3.84 ± 3.37	5.74 ± 3.57	6.14 ± 3.54	4.82 ± 3.46
**Enabling factors**						
**Income quintile, n(%)**						
Missing	73 (0.2%)	41 (0.2%)	32 (0.3%)	193 (0.2%)	142 (0.2%)	51 (0.1%)
Q1 (lowest)	11,973 (33.4%)	7,832 (33.7%)	4,141 (33.0%)	29,925 (25.4%)	20,313 (24.7%)	9,612 (27.2%)
Q2	7,737 (21.6%)	4,969 (21.4%)	2,768 (22.1%)	25,243 (21.5%)	17,564 (21.3%)	7,679 (21.7%)
Q3	6,219 (17.4%)	4,075 (17.5%)	2,144 (17.1%)	22,310 (19.0%)	15,603 (19.0%)	6,707 (19.0%)
Q4	5,222 (14.6%)	3,385 (14.5%)	1,837 (14.6%)	20,641 (17.5%)	14,653 (17.8%)	5,988 (16.9%)
Q5 (highest)	4,432 (12.4%)	2,873 (12.3%)	1,559 (12.4%)	19,027 (16.2%)	13,834 (16.8%)	5,193 (14.7%)
**Rurality, n(%)**						
Missing	73 (0.2%)	41 (0.2%)	32 (0.3%)	193 (0.2%)	142 (0.2%)	51 (0.1%)
Urban	32,708 (91.3%)	21,550 (92.6%)	11,158 (88.9%)	101,932 (86.6%)	73,200 (89.0%)	28,732 (81.3%)
Rural	3,038 (8.5%)	1,683 (7.2%)	1,355 (10.8%)	15,513 (13.2%)	8,939 (10.9%)	6,574 (18.6%)
**Usual provider of care (% yes)**	32,423 (90.5%)	22,421 (96.3%)	10,002 (79.7%)	112,375 (95.5%)	81,393 (98.9%)	30,982 (87.6%)
**Need factors**						
**Self-harm diagnosis at index ED visit, n(%)**	175 (0.5%)	114 (0.5%)	61 (0.5%)	2,376 (2.0%)	1,660 (2.0%)	716 (2.0%)
**Acuity (CTAS) score at index ED visit, n(%)**						
High acuity	32,470 (90.7%)	21,029 (90.4%)	11,441 (91.2%)	100,608 (85.5%)	71,320 (86.7%)	29,288 (82.8%)
Low acuity	3,256 (9.1%)	2,188 (9.4%)	1,068 (8.5%)	16,662 (14.2%)	10,735 (13.0%)	5,927 (16.8%)

© 2021 Kurdyak P. *JAMA Network Open.*

**Admission to inpatient care from index ED visit, n(%)**	22,747 (63.5%)	14,417 (61.9%)	8,330 (66.4%)	34,773 (29.6%)	25,937 (31.5%)	8,836 (25.0%)
**Death during index ED visit, n(%)**	< 6	< 6	0 (0.0%)	< 6	< 6	< 6

© 2021 Kurdyak P. *JAMA Network Open.*

eTable 3. Characteristics of adults with a first contact emergency department visit for anxiety or substance and substance-related disorders, by prior outpatient care for mental health and addictions-related disorders, 2010-2018 (N=403,877).

	Anxiety and Adustment Disorders			Substance-related Disorders		
**Variable**	**Total**	**Prio Contact**	**First Contact**	**Total**	**Prior Contact**	**First Contact**
**Adults with a first contact emergency department**	**251,236**	**132,757**	**118,479**	**152,641**	**67,389**	**85,252**
**Predisposing factors**						
**Age, mean ± SD**	40.86 ± 18.82	40.92 ± 18.32	40.78 ± 19.37	36.48 ± 17.45	38.33 ± 16.51	35.01 ± 18.02
**Age group (years), n(%)**						
16-24	62,352 ((24.8%)	31,382 (23.6%)	30,970 (26.1%)	54,655 (35.8%)	18,126 (26.9%)	36,529 (42.8%)
25-34	49,533 (19.7%)	25,833 (19.5%)	23,700 (20.0%)	29,438 (19.3%)	14,483 (21.5%)	14,955 (17.5%)
35-44	41,736 (16.6%)	23,241 (17.5%)	18,495 (15.6%)	19,336 (12.7%)	10,772 (16.0%)	8,564 (10.0%)
45-54	39,011 (15.5%)	21,907 (16.5%)	17,104 (14.4%)	21,614 (14.2%)	11,668 (17.3%)	9,946 (11.7%)
55-64	26,031 (10.4%)	14,399 (10.8%)	11,632 (9.8%)	15,418 (10.1%)	7,475 (11.1%)	7,943 (9.3%)
65-84	27,414 (10.9%)	13,740 (10.3%)	13,674 (11.5%)	11,186 (7.3%)	4,498 (6.7%)	6,688 (7.8%)
85+	5,159 (2.1%)	2,255 (1.7%)	2,904 (2.5%)	994 (0.7%)	367 (0.5%)	627 (0.7%)
**Sex, n(%)**						
Female	149,148 (59.4%)	83,817 (63.1%)	65,331 (55.1%)	54,678 (35.8%)	26,825 (39.8%)	27,853 (32.7%)
Male	102,088 (40.6%)	48,940 (36.9%)	53,148 (44.9%)	97,963 (64.2%)	40,564 (60.2%)	57,399 (67.3%)
**Immigration category, n(%)**						
Non-immigrant	220,716 (87.9%)	117,206 (88.3%)	103,510 (87.4%)	136,250 (89.3%)	61,461 (91.2%)	74,789 (87.7%)
Family class immigrants	10,954 (4.4%)	5,645 (4.3%)	5,309 (4.5%)	5,282 (3.5%)	2,072 (3.1%)	3,210 (3.8%)
Resettled refugees	6,804 (2.7%)	3,503 (2.6%)	3,301 (2.8%)	4,418 (2.9%)	1,647 (2.4%)	2,771 (3.3%)
Other immigrants	579 (0.2%)	279 (0.2%)	300 (0.3%)	299 (0.2%)	109 (0.2%)	190 (0.2%)
Economic class immigrants	12,183 (4.8%)	6,124 (4.6%)	6,059 (5.1%)	6,392 (4.2%)	2,100 (3.1%)	4,292 (5.0%)
**Number of comorbid conditions, mean ± SD**	6.19 ± 3.62	6.80 ± 3.63	5.51 ± 3.50	5.26 ± 3.44	6.20 ± 3.52	4.52 ± 3.20
**Enabling factors**						
**Income quintile, n(%)**						
Missing	3,274 (1.3%)	1,140 (0.9%)	2,134 (1.8%)	4,056 (2.7%)	1,204 (1.8%)	2,852 (3.3%)
Q1 (lowest)	63,420 (25.2%)	33,392 (25.2%)	30,028 (25.3%)	43,241 (28.3%)	19,513 (29.0%)	23,728 (27.8%)
Q2	53,303 (21.2%)	28,008 (21.1%)	25,295 (21.3%)	31,446 (20.6%)	14,196 (21.1%)	17,250 (20.2%)
Q3	48,842 (19.4%)	25,635 (19.3%)	23,207 (19.6%)	27,442 (18.0%)	11,974 (17.8%)	15,468 (18.1%)
Q4	45,149 (18.0%)	23,997 (18.1%)	21,152 (17.9%)	24,897 (16.3%)	10,701 (15.9%)	14,196 (16.7%)
Q5 (highest)	39,399 (15.7%)	21,234 (16.0%)	18,165 (15.3%)	24,236 (15.9%)	10,547 (15.7%)	13,689 (16.1%)
**Rurality, n(%)**						
Missing	524 (0.3%)	181 (0.3%)	343 (0.4%)	94 (0.2%)	63 (0.2%)	31 (0.1%)
Urban	207,424 (82.6%)	114,071 (85.9%)	93,353 (78.8%)	134,056 (87.8%)	60,353 (89.6%)	73,703 (86.5%)
Rural	43,493 (17.3%)	18,513 (13.9%)	24,980 (21.1%)	18,061 (11.8%)	6,855 (10.2%)	11,206 (13.1%)
**Usual provider of care (% yes)**	239,624 (95.4%)	131,681 (99.2%)	107,943 (91.1%)	139,773 (91.6%)	66,265 (98.3%)	73,508 (86.2%)
**Need factors**						
**Self-harm diagnosis at index ED visit, n(%)**	1,785 (0.7%)	1,053 (0.8%)	732 (0.6%)	936 (0.6%)	531 (0.8%)	405 (0.5%)

© 2021 Kurdyak P. *JAMA Network Open.*

**Acuity (CTAS) score at index ED visit, n(%)**						
High acuity	193,311 (76.9%)	104,559 (78.8%)	88,752 (74.9%)	131,505 (86.2%)	57,930 (86.0%)	73,575 (86.3%)
Low acuity	57,215 (22.8%)	27,901 (21.0%)	29,314 (24.7%)	20,752 (13.6%)	9,321 (13.8%)	11,431 (13.4%)
**Admission to inpatient care from index ED visit,**	7,924 (3.2%)	5,228 (3.9%)	2,696 (2.3%)	11,517 (7.5%)	6,164 (9.1%)	5,353 (6.3%)
**Death during index ED visit, n(%)**	9 (0.0%)	6 (0.0%)	< 6	23 (0.0%)	8 (0.0%)	15 (0.0%)

© 2021 Kurdyak P. *JAMA Network Open.*

eTable 4. Characteristics of adults with a first contact emergency department visit, by prior outpatient care for mental health and addictions-related disorders and presence or absence of self-harm diagnosis, 2010-2018 (N=659,084).

Variable	Total	Prior Contact	First Contact	Total	Prior Contact	First Contact
**Adults with a first contact emergency department visit, N**	**56,164**	**31,271**	**24,893**	**602,920**	**328,889**	**274,031**
**Predisposing factors**						
**Age, mean ± SD**	37.30 ± 17.99	37.85 ± 17.32	36.60 ± 18.78	39.30 ± 18.49	40.06 ± 17.93	38.39 ± 19.10
**Age group (years), n(%)**						
16-24	18,982 (33.8%)	9,643 (30.8%)	9,339 (37.5%)	174,006 (28.9%)	83,568 (25.4%)	90,438 (33.0%)
25-34	9,981 (17.8%)	5,440 (17.4%)	4,541 (18.2%)	115,460 (19.2%)	63,541 (19.3%)	51,919 (18.9%)
35-44	8,085 (14.4%)	4,949 (15.8%)	3,136 (12.6%)	91,752 (15.2%)	54,795 (16.7%)	36,957 (13.5%)
44-54	8,882 (15.8%)	5,628 (18.0%)	3,254 (13.1%)	92,411 (15.3%)	55,841 (17.0%)	36,570 (13.3%)
55-64	5,528 (9.8%)	3,361 (10.7%)	2,167 (8.7%)	62,682 (10.4%)	36,653 (11.1%)	26,029 (9.5%)
65-84	3,942 (7.0%)	1,935 (6.2%)	2,007 (8.1%)	57,021 (9.5%)	30,049 (9.1%)	26,972 (9.8%)
85+	764 (1.4%)	315 (1.0%)	449 (1.8%)	9,588 (1.6%)	4,442 (1.4%)	5,146 (1.9%)
**Sex, n(%)**						
Female	30,628 (54.5%)	18,783 (60.1%)	11,845 (47.6%)	309,731 (51.4%)	183,146 (55.7%)	126,585 (46.2%)
Male	25,536 (45.5%)	12,488 (39.9%)	13,048 (52.4%)	293,189 (48.6%)	145,743 (44.3%)	147,446 (53.8%)
**Immigration category, n(%)**						
Non-immigrant	49,442 (88.0%)	28,193 (90.2%)	21,249 (85.4%)	533,181 (88.4%)	292,932 (89.1%)	240,249 (87.7%)
Family class immigrants	2,466 (4.4%)	1,119 (3.6%)	1,347 (5.4%)	24,522 (4.1%)	12,951 (3.9%)	11,571 (4.2%)
Resettled refugees	1,585 (2.8%)	733 (2.3%)	852 (3.4%)	16,309 (2.7%)	8,366 (2.5%)	7,943 (2.9%)
Other immigrants	123 (0.2%)	53 (0.2%)	70 (0.3%)	1,332 (0.2%)	651 (0.2%)	681 (0.2%)
Economic class immigrants	2,548 (4.5%)	1,173 (3.8%)	1,375 (5.5%)	27,576 (4.6%)	13,989 (4.3%)	13,587 (5.0%)
**Number of comorbid conditions, mean ± SD**	6.12 ± 3.52	6.73 ± 3.50	5.37 ± 3.38	5.78 ± 3.60	6.40 ± 3.62	5.05 ± 3.44
**Enabling factors**						
**Income quintile, n(%)**						
Missing	336 (0.6%)	140 (0.4%)	196 (0.8%)	3,419 (0.6%)	1,495 (0.5%)	1,924 (0.7%)
Q1 (lowest)	15,249 (27.2%)	8,384 (26.8%)	6,865 (27.6%)	160,029 (26.5%)	86,947 (26.4%)	73,082 (26.7%)
Q2	12,008 (21.4%)	6,674 (21.3%)	5,334 (21.4%)	127,346 (21.1%)	69,597 (21.2%)	57,749 (21.1%)
Q3	10,395 (18.5%)	5,786 (18.5%)	4,609 (18.5%)	113,481 (18.8%)	61,632 (18.7%)	51,849 (18.9%)
Q4	9,563 (17.0%)	5,375 (17.2%)	4,188 (16.8%)	103,959 (17.2%)	56,790 (17.3%)	47,169 (17.2%)
Q5 (highest)	8,613 (15.3%)	4,912 (15.7%)	3,701 (14.9%)	94,686 (15.7%)	52,428 (15.9%)	42,258 (15.4%)
**Rurality, n(%)**						
Missing	94 (0.2%)	63 (0.2%)	31 (0.1%)	1,242 (0.2%)	610 (0.2%)	632 (0.2%)
Urban	49,087 (87.4%)	27,934 (89.3%)	21,153 (85.0%)	514,377 (85.3%)	289,421 (88.0%)	224,956 (82.1%)
Rural	6,983 (12.4%)	3,274 (10.5%)	3,709 (14.9%)	87,301 (14.5%)	38,858 (11.8%)	48,443 (17.7%)
**Usual provider of care (% yes)**	52,912 (94.2%)	30,835 (98.6%)	22,077 (88.7%)	567,350 (94.1%)	324,660 (98.7%)	242,690 (88.6%)
**Need factors**						
**MHA diagnosis at index ED visit**						
Substance related disorders	936 (1.7%)	531 (1.7%)	405 (1.6%)	151,705 (25.2%)	66,858 (20.3%)	84,847 (31.0%)
Schizophrenia, delusional, and non-organic psychotic	175 (0.3%)	114 (0.4%)	61 (0.2%)	35,644 (5.9%)	23,160 (7.0%)	12,484 (4.6%)
Mood and affective disorders	2,376 (4.2%)	1,660 (5.3%)	716 (2.9%)	115,262 (19.1%)	80,621 (24.5%)	34,641 (12.6%)
Anxiety and adjustment disorders	1,785 (3.2%)	1,053 (3.4%)	732 (2.9%)	249,451 (41.4%)	131,704 (40.0%)	117,747 (43.0%)
Other mental health disorders	50,892 (90.6%)	27,913 (89.3%)	22,979 (92.3%)	50,858 (8.4%)	26,546 (8.1%)	24,312 (8.9%)
**Acuity (CTAS) score at index ED visit, n(%)**						
High acuity	49,801 (88.7%)	29,200 (93.4%)	20,601 (82.8%)	493,667 (81.9%)	273,647 (83.2%)	220,020 (80.3%)
Low acuity	6,251 (11.1%)	2,023 (6.5%)	4,228 (17.0%)	107,587 (17.8%)	54,478 (16.6%)	53,109 (19.4%)
**Admission to inpatient care from index ED visit, n(%)**	22,722 (40.5%)	14,177 (45.3%)	8,545 (34.3%)	79,864 (13.2%)	53,507 (16.3%)	26,357 (9.6%)

© 2021 Kurdyak P. *JAMA Network Open.*

**Death during index ED visit, n(%)**	465 (0.8%)	226 (0.7%)	239 (1.0%)	41 (0.0%)	19 (0.0%)	22 (0.0%)

© 2021 Kurdyak P. *JAMA Network Open.*

eTable 5. Adjusted odds ratios of no prior outpatient care for mental health and addictions-related emergency department visits, by diagnostic category, 2010-2018 (n=659,084).

Variable	aOR (95% CI)					
	Schizophrenia and Other Non-Organic Psychotic disorders	Mood Disorders	Anxiety and adjustment disorders	Substance-related disorders	Any self-harm	No self-harm
**Model 1: Predisposing factors**						
**Age (reference = 16-24 years old)**						
25-34	0.79 (0.76-0.82)	0.79 (0.76-0.82)	0.99 (0.97-1.00)	0.77 (0.76-0.78)	0.91 (0.89-0.93)	0.88 (0.87-0.89)
35-44	0.73 (0.70-0.77)	0.73 (0.70-0.77)	0.93 (0.92-0.94)	0.69 (0.68-0.70)	0.80 (0.78-0.83)	0.81 (0.80-0.81)
45-54	0.74 (0.71-0.78)	0.74 (0.71-0.78)	0.94 (0.93-0.95)	0.73 (0.72-0.75)	0.80 (0.78-0.83)	0.81 (0.81-0.82)
55-64	0.76 (0.72-0.80)	0.76 (0.72-0.80)	1.01 (0.99-1.02)	0.85 (0.84-0.87)	0.90 (0.87-0.93)	0.89 (0.88-0.90)
65-84	1.04 (0.99-1.10)	1.04 (0.99-1.10)	1.25 (1.23-1.27)	1.08 (1.06-1.10)	1.30 (1.25-1.35)	1.13 (1.12-1.14)
85+	1.54 (1.42-1.68)	1.54 (1.42-1.68)	1.50 (1.46-1.54)	1.28 (1.22-1.34)	1.61 (1.51-1.71)	1.38 (1.36-1.41)
**Male (reference = female)**	1.01 (0.98-1.04)	1.01 (0.98-1.04)	1.10 (1.09-1.11)	1.08 (1.07-1.09)	1.23 (1.21-1.26)	1.13 (1.12-1.14)
**Immigration category (reference = non-immigrant)**						
Economic class immigrants	1.12 (1.06-1.18)	1.12 (1.06-1.18)	1.12 (1.10-1.14)	1.18 (1.16-1.20)	1.29 (1.24-1.33)	1.13 (1.11-1.14)
Other immigrants	1.26 (1.04-1.53)	1.26 (1.04-1.53)	1.20 (1.12-1.30)	1.15 (1.06-1.25)	1.36 (1.17-1.59)	1.20 (1.14-1.26)
Resettled refugees	1.12 (1.05-1.20)	1.12 (1.05-1.20)	1.11 (1.08-1.14)	1.18 (1.16-1.21)	1.31 (1.26-1.37)	1.14 (1.12-1.16)
Family class immigrants	1.14 (1.08-1.20)	1.14 (1.08-1.20)	1.12 (1.10-1.14)	1.18 (1.15-1.21)	1.35 (1.31-1.40)	1.13 (1.12-1.15)
**Number of comorbid conditions (per each additional condition)**	0.92 (0.91-0.92)	0.92 (0.91-0.92)	0.94 (0.94-0.94)	0.94 (0.94-0.94)	0.94 (0.93-0.94)	0.94 (0.94-0.94)
**Model 2: Predisposing + enabling factors**						
**Age (reference = 16-24 years old)**						
25-34	0.77 (0.74-0.80)	0.86 (0.84-0.88)	0.97 (0.96-0.98)	0.75 (0.74-0.76)	0.90 (0.87-0.92)	0.86 (0.85-0.87)
35-44	0.70 (0.67-0.73)	0.73 (0.71-0.75)	0.91 (0.90-0.92)	0.67 (0.66-0.68)	0.80 (0.77-0.82)	0.79 (0.78-0.79)
45-54	0.69 (0.67-0.73)	0.67 (0.65-0.69)	0.92 (0.91-0.93)	0.72 (0.71-0.73)	0.80 (0.78-0.82)	0.79 (0.78-0.80)
55-64	0.70 (0.66-0.73)	0.70 (0.68-0.73)	0.98 (0.96-0.99)	0.84 (0.82-0.85)	0.90 (0.86-0.93)	0.86 (0.85-0.87)
65-84	0.95 (0.90-1.00)	0.91 (0.88-0.94)	1.21 (1.19-1.23)	1.05 (1.04-1.07)	1.29 (1.24-1.33)	1.08 (1.07-1.10)
85+	1.38 (1.26-1.50)	1.39 (1.30-1.49)	1.45 (1.41-1.48)	1.24 (1.18-1.31)	1.59 (1.49-1.69)	1.33 (1.30-1.35)
**Male (reference = female)**	0.99 (0.96-1.02)	1.20 (1.18-1.22)	1.09 (1.08-1.10)	1.07 (1.06-1.08)	1.21 (1.19-1.24)	1.11 (1.11-1.12)
**Immigration category (reference = non-immigrant)**						
Economic class immigrants	1.18 (1.12-1.25)	1.04 (0.99-1.09)	1.18 (1.16-1.20)	1.22 (1.20-1.24)	1.34 (1.30-1.39)	1.19 (1.17-1.20)
Other immigrants	1.28 (1.06-1.54)	1.18 (0.97-1.45)	1.25 (1.15-1.35)	1.17 (1.07-1.27)	1.44 (1.23-1.67)	1.24 (1.18-1.30)
Resettled refugees	1.15 (1.08-1.23)	1.10 (1.04-1.17)	1.17 (1.14-1.20)	1.21 (1.18-1.24)	1.35 (1.29-1.41)	1.19 (1.17-1.21)
Family class immigrants	1.18 (1.12-1.25)	1.17 (1.12-1.23)	1.18 (1.15-1.20)	1.21 (1.18-1.23)	1.40 (1.35-1.45)	1.19 (1.17-1.20)
**Number of comorbid conditions (per each additional condition)**	0.95 (0.94-0.95)	0.95 (0.95-0.95)	0.95 (0.95-0.95)	0.95 (0.95-0.95)	0.95 (0.95-0.95)	0.95 (0.95-0.96)
**Income quintile (reference = Q5: highest)**						
Q1 (lowest)	0.98 (0.93-1.02)	1.18 (1.15-1.21)	1.01 (1.00-1.03)	0.99 (0.97-1.00)	1.03 (1.00-1.06)	1.01 (1.00-1.02)
Q2	1.02 (0.97-1.07)	1.13 (1.09-1.16)	1.03 (1.01-1.04)	1.00 (0.98-1.01)	1.03 (1.00-1.07)	1.02 (1.01-1.03)
Q3	0.99 (0.94-1.04)	1.11 (1.08-1.15)	1.03 (1.01-1.04)	1.01 (1.00-1.03)	1.04 (1.00-1.07)	1.03 (1.02-1.04)
Q4	1.01 (0.96-1.07)	1.08 (1.04-1.11)	1.02 (1.01-1.03)	1.02 (1.00-1.03)	1.02 (0.99-1.06)	1.02 (1.01-1.03)
**Rural (reference = urban)**	1.25 (1.19-1.30)	1.40 (1.38-1.43)	1.24 (1.23-1.25)	1.10 (1.09-1.12)	1.17 (1.14-1.20)	1.24 (1.24-1.25)
**No UPC (reference = has usual provider of care)**	2.08 (2.02-2.15)	2.33 (2.29-2.38)	1.65 (1.64-1.67)	1.50 (1.48-1.51)	1.67 (1.64-1.71)	1.71 (1.70-1.72)
**Model 3: Predisposing + enabling + need factors**						
**Age (reference = 16-24 years old)**						
25-34	0.77 (0.74-0.80)	0.85 (0.83-0.87)	0.96 (0.95-0.98)	0.76 (0.75-0.76)	0.89 (0.87-0.91)	0.86 (0.86-0.87)
35-44	0.70 (0.67-0.73)	0.73 (0.71-0.75)	0.91 (0.90-0.92)	0.68 (0.67-0.69)	0.80 (0.78-0.83)	0.80 (0.79-0.80)
45-54	0.70 (0.67-0.73)	0.69 (0.67-0.71)	0.92 (0.91-0.93)	0.72 (0.71-0.74)	0.82 (0.79-0.84)	0.81 (0.80-0.81)
55-64	0.70 (0.66-0.74)	0.72 (0.70-0.74)	0.98 (0.96-0.99)	0.85 (0.83-0.86)	0.92 (0.89-0.95)	0.89 (0.88-0.89)
65-84	0.95 (0.90-1.00)	0.93 (0.90-0.96)	1.21 (1.19-1.23)	1.07 (1.05-1.09)	1.33 (1.28-1.37)	1.12 (1.11-1.13)
85+	1.38 (1.27-1.51)	1.42 (1.33-1.51)	1.45 (1.41-1.49)	1.27 (1.21-1.33)	1.63 (1.53-1.73)	1.37 (1.34-1.40)
**Male (reference = female)**	0.99 (0.96-1.02)	1.21 (1.18-1.23)	1.09 (1.08-1.10)	1.07 (1.06-1.08)	1.17 (1.15-1.19)	1.12 (1.11-1.13)

© 2021 Kurdyak P. *JAMA Network Open.*

**Immigration category (reference = non-immigrant)**						
Economic class immigrants	1.18 (1.12-1.24)	1.05 (1.00-1.09)	1.18 (1.16-1.20)	1.21 (1.19-1.23)	1.32 (1.27-1.37)	1.19 (1.17-1.20)
Other immigrants	1.28 (1.06-1.55)	1.19 (0.98-1.46)	1.25 (1.16-1.35)	1.16 (1.06-1.26)	1.45 (1.24-1.68)	1.25 (1.19-1.31)
Resettled refugees	1.15 (1.08-1.23)	1.11 (1.05-1.18)	1.17 (1.14-1.20)	1.21 (1.18-1.23)	1.33 (1.27-1.39)	1.20 (1.18-1.21)
Family class immigrants	1.18 (1.12-1.24)	1.18 (1.13-1.24)	1.18 (1.16-1.20)	1.21 (1.18-1.23)	1.38 (1.33-1.44)	1.19 (1.18-1.21)
**Number of comorbid conditions (per each additional condition)**	0.95 (0.94-0.95)	0.95 (0.95-0.95)	0.95 (0.95-0.95)	0.95 (0.95-0.95)	0.95 (0.95-0.96)	0.95 (0.95-0.95)
**Income quintile (reference = Q5: highest)**						
Q1 (lowest)	0.98 (0.93-1.02)	1.17 (1.14-1.20)	1.01 (1.00-1.03)	0.99 (0.97-1.00)	1.04 (1.01-1.07)	1.01 (1.00-1.02)
Q2	1.02 (0.97-1.07)	1.12 (1.09-1.15)	1.02 (1.01-1.04)	1.00 (0.98-1.01)	1.04 (1.01-1.07)	1.02 (1.01-1.03)
Q3	0.99 (0.94-1.04)	1.11 (1.08-1.14)	1.03 (1.01-1.04)	1.01 (1.00-1.03)	1.04 (1.01-1.08)	1.03 (1.02-1.04)
Q4	1.01 (0.96-1.07)	1.07 (1.04-1.11)	1.02 (1.00-1.03)	1.02 (1.00-1.03)	1.03 (1.00-1.06)	1.02 (1.01-1.03)
**Rural (reference = urban)**	1.25 (1.20-1.30)	1.36 (1.33-1.39)	1.22 (1.21-1.24)	1.11 (1.09-1.12)	1.15 (1.12-1.18)	1.22 (1.21-1.22)
**No UPC (reference = has usual provider of care)**	2.08 (2.02-2.15)	2.33 (2.29-2.38)	1.65 (1.64-1.67)	1.50 (1.48-1.51)	1.67 (1.63-1.70)	1.72 (1.71-1.73)
**High acuity index ED visit (reference = low acuity)**	1.05 (1.00-1.11)	0.93 (0.91-0.95)	0.95 (0.94-0.96)	1.04 (1.03-1.05)	0.71 (0.70-0.73)	0.97 (0.96-0.97)
**Admitted from ED (reference = not admitted)**	1.06 (1.03-1.10)	0.83 (0.81-0.85)	0.72 (0.70-0.74)	0.86 (0.84-0.88)	0.87 (0.85-0.89)	0.69 (0.68-0.69)
**Self-harm diagnosis at index ED visit**	1.01 (0.83-1.23)	1.11 (1.05-1.18)	0.94 (0.89-0.99)	0.82 (0.77-0.88)	n/a	n/a

© 2021 Kurdyak P. *JAMA Network Open.*
